# Quarantine Vehicle Scheduling for Transferring High-Risk Individuals in Epidemic Areas

**DOI:** 10.3390/ijerph17072275

**Published:** 2020-03-27

**Authors:** Min-Xia Zhang, Hong-Fan Yan, Jia-Yu Wu, Yu-Jun Zheng

**Affiliations:** 1 College of Computer Science and Technology, Zhejiang University of Technology, Hangzhou 310023, China; minxia_zhang@yeah.net (M.-X.Z.); hongfangyan@compintell.cn (H.-F.Y.); jiayuwu@compintell.cn (J.-Y.W.); 2 School of Information Science and Engineering, Hangzhou Normal University, Hangzhou 311121, China

**Keywords:** public health emergencies, epidemics, medical isolation, vehicle scheduling, optimization, water wave optimization (WWO)

## Abstract

In a large-scale epidemic outbreak, there can be many high-risk individuals to be transferred for medical isolation in epidemic areas. Typically, the individuals are scattered across different locations, and available quarantine vehicles are limited. Therefore, it is challenging to efficiently schedule the vehicles to transfer the individuals to isolated regions to control the spread of the epidemic. In this paper, we formulate such a quarantine vehicle scheduling problem for high-risk individual transfer, which is more difficult than most well-known vehicle routing problems. To efficiently solve this problem, we propose a hybrid algorithm based on the water wave optimization (WWO) metaheuristic and neighborhood search. The metaheuristic uses a small population to rapidly explore the solution space, and the neighborhood search uses a gradual strategy to improve the solution accuracy. Computational results demonstrate that the proposed algorithm significantly outperforms several existing algorithms and obtains high-quality solutions on real-world problem instances for high-risk individual transfer in Hangzhou, China, during the peak period of the novel coronavirus pneumonia (COVID-19).

## 1. Introduction

**Figure 1:**
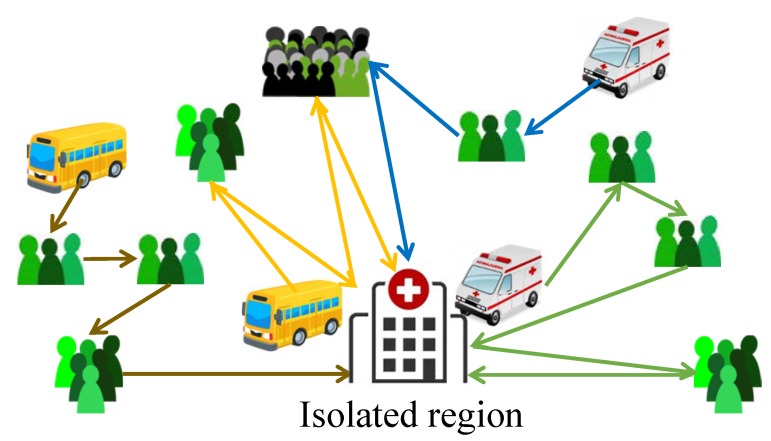
An illustration of the vehicle routing problem for transferring high-risk individuals in epidemics.

In an outbreak of a severe epidemic, a large number of suspected cases and close contacts of patients, which we call *high-risk individuals*, need to be isolated for medical observation as soon as possible to prevent the spread of the virus carried by them. Another benefit of isolation is to reduce the mortality, that is, whenever a high-risk individual is diagnosed, he/she can get prompt treatment. However, high-risk individuals are often scattered in a city, and the number of available quarantine vehicles that are eligible to transfer high-risk individuals is limited. Therefore, we must efficiently schedule the vehicles, i.e., assign high-risk individuals to the vehicles and determine the route of each vehicle (as illustrated by Figure 1), to transfer the individuals to isolated regions as quickly as possible to control the spread of the epidemic.

The considered problem can be regarded as a variant of the well-known vehicle routing problem (VRP) [1], which has been widely studied in the fields of computer science and operations research. Nevertheless, This problem differs from most well-known VRPs in the following aspects:

- The number of high-risk individuals in a location or small area may exceed the capacity of a vehicle. In such a case, it may require a vehicle to go to the area more than once, or require two or more vehicles to load the individuals in the area.
- After loading high-risk individuals in an area, if a vehicle still has room, it can either go to another area to load more individuals or directly go back to the isolated region (some individuals in serious condition should be immediately sent to the hospital and are excluded from the individuals to be transferred).
- The objective of the problem is to reduce the risk of epidemic spread as much as possible, and hence the objective function should be defined based on the exposure durations of high-risk individuals.

Therefore, this problem is significantly more complex than most existing VRPs that are known to be NP-hard. For large instances of the problem, exact algorithms (such as branch-and-bound) are often impractical, and traditional metaheuristic algorithms (such as genetic algorithms) also converge slowly [2]. To handle this difficult problem, we propose a hybrid water wave optimization (WWO) [3] and neighborhood search algorithm, which adapts the WWO metaheuristic to efficiently explore the solution space of the problem and utilizes neighborhood search to improve the solution accuracy. Compared to most existing metaheuristics that require many generations to evolve a large population to explore the solution space, the proposed WWO algorithm evolves a small population of solutions to rapidly locate an optimal or near-optimal solution. The neighborhood search method uses a gradual strategy to further improve the solution accuracy. We demonstrate the efficiency of the proposed algorithm on real-world instances for transferring thousands of high-risk individuals during the peak period of the novel coronavirus pneumonia (COVID-19) in Hangzhou city, China. The main contributions of this paper can be summarized as follows:

- We present a new VRP variant based on the requirements of high-risk individual isolation in large-scale epidemics.
- We provide a set of rules for scheduling multiple vehicles for high-risk individual transfer.
- We propose an efficient algorithm for the problem, which was used to solve real-world instances in COVID-19 within a short response time.

The remainder of this paper is structured as follows. Section 2 introduces related work on basic and emergency VRPs, Section 3 formulates the quarantine VRP for transferring high-risk individuals in epidemics, which also provides the basic scheduling rules that are useful in practice. Section 4 proposes the hybrid algorithm, Section 5 presents the computational results, and Section 6 concludes with a discussion.

## 2. Related Work

First proposed by Dantzig and Ramse [4], VRPs are well-known NP-hard problems that have been widely studied in the fields of computer science and operations research [1]. Traditional exact methods are only applicable to small problem instances [5]. In recent years, considerable efforts have been devoted to evolutionary algorithms [6,7,8], including genetic algorithms (GA) [9,10,11,12,13], particle swarm optimization [14,15,16,17,18], ant colony optimization (ACO) [19,20,21,22,23], artificial bee colony [24,25,26], biogeography-based optimization [27,28], etc., for VRPs. Compared to exact algorithms and construction heuristics, evolutionary algorithms are more capable of jumping out of local optima and obtaining optimal or near-optimal solutions within an acceptable time by evolving a population of candidate solutions to simultaneously explore multiple regions of the search space [29].

Emergency VRPs are more challenging because they are needed to be solved within a limited response time [30]. Daskin and Haghani [31] studied an emergency VRP that extends the basic VRP to an emergency scene by allowing edge travel times to be normally distributed and the path travel times of different vehicles to be correlated. In [32] Haghani et al. evaluated different response strategies including first-called first-served, nearest-origin assignment, and flexible assignment for assigning response vehicles and guiding those vehicles through non-congested routes. Vargas-Villamil [33] used mixed-integer nonlinear programming to solve a vehicle routing and dispatching problem for emergency personnel evacuation from off-shore oil platforms. Wang and Deng [34] studied an emergency VRP in post-disaster, where customer demands are described as the fuzzy variables; they proposed a two-stage approach, where the first stage optimizes the vehicle routes to minimize transportation time, and the second stage uses fuzzy linear programming to minimize transportation cost. Wohlgemuth et al. [35] considered a dynamic VRP with varying travel times and unknown orders in the specific environment of emergencies; they used a multi-stage mixed integer programming method to find solutions that decrease delays and increase equipment utilization. Chini et al. [36] studied a VRP in both deterministic and stochastic scenarios; they proposed a routing algorithm for graph-represented mission spaces and a switching algorithm to dynamically change the vehicle behavior according to the time-variable configuration of both vehicles and targets. Ye et al. [37] studied an emergency evacuation path planning problem in catastrophic events, which was modeled using an improved cellular automaton based on ACO. Considering fire evacuation scenarios, Shahparvari and Abbasi [38] proposed a greedy heuristic method to solve a VRP under uncertainties in evacuee population, time windows, and bushfire propagation; they applied their approach to a real case study of the 2009 Black Saturday bushfires in Victoria, Australia.

Exact algorithms are not practical for solving large instances of emergency VRPs. Moreover, traditional evolutionary algorithms also converge slowly in large search spaces, and therefore studies on adapting evolutionary algorithms for emergency VRPs are relatively few. Sun et al. [39] proposed an improved ACO algorithm to solve an emergency VRP, where GA is utilized to optimize the parameters of ACO. Chen [40] utilized a GA to solve an emergency VRP with strict timeliness requirements. Du and Yi [41] also tested the performance of GAs for a multi-objective emergency VRP. Nevertheless, the results showed that classical GAs only perform well on small or medium instances. To solve emergency transportation that combines cumulative VRP and multi-depot VRP, Wang et al. [42] proposed an improved ACO algorithm with a smart design of the ant tabus to speed up solution construction. Recently, Fallahi and Sefrioui [43] studied a problem of scheduling ambulances to bring the maximal number of alive victims to hospitals; they proposed a memetic algorithm that employs variable neighborhood search to improve solution accuracy. However, to the best of our knowledge, there have been no reports of VRPs for transferring high-risk individuals in epidemic areas, mainly due to the specific characteristics of the problem in the epidemic situation.

## 3. Problem Description

### 3.1. Problem Inputs

We consider the problem of routing multiple quarantine vehicles to transfer high-risk individuals (potential propagators) to an isolated region in epidemic situations. Let $A\;=\;\{a_{1},a_{2},\ldots ,a_{n}\}$ be the set of *n* locations or small areas with high-risk individuals, and $b_{j}$ be the number of high-risk individuals in $a_{j}$ (1≤j≤n). We have a set $V\;=\;\{v_{1},v_{2},\ldots ,v_{m}\}$ of vehicles, and the capacity of $v_{i}$ (i.e., the maximum number of individuals that can be carried by $v_{i}$) is $c_{i}$ (1≤i≤m). Based on the distances between the areas and the velocities of the vehicles, we can obtain the travel time $t_{i,j}$ of each vehicle $v_{i}$ from its original location to each area $a_{j}$, the time $t_{i,j,j^{\prime }}$ for $v_{i}$ to travel from $a_{j}$ to $a_{j^{\prime }}$, and the time $t_{i,j}^{\prime }$ for $v_{i}$ to travel from $a_{j}$ to the isolated region ($1\;\leq \;i\;\leq \;m;1\;\leq \;j,j^{\prime }\;\leq \;n$). We also have the average time interval $\Delta t_{i,j}$ between loading two individuals in $a_{j}$ by $v_{i}$, which depends on the dispersion of individuals in the area (for example, if individuals are gathered in a community health center, the interval can be several seconds; if individuals are in different units of a housing estate, the interval can be several minutes). The inputs indicate that we can use vehicles with different capacities and velocities, and thus the problem is a heterogeneous VRP. Table 1 lists the variables used in the problem formulation.

### 3.2. Decision Variables and Scheduling Rules

The problem needs to first determine the sequence $x_{i}\;=\;\left\{x_{i,1},x_{i,2},\ldots ,x_{i,n_{i}}\right\}$ of areas to be visited by each vehicle $v_{i}$, where $x_{i,j}$ is the index of $v_{i}$’s *j*th visiting area in *A*, and $n_{i}$ is the number of areas assigned to $v_{i}$. Moreover, as aforementioned, after visiting an area, a vehicle may either go back to the isolated region or go to the next area, so we also need to determine the sequence $y_{i}\;=\;\left\{y_{i,1},y_{i,2},\ldots ,y_{i,n_{i}\;-\;1}\right\}$ of intermediate decisions of $v_{i}$, where $y_{i,j}\;=\;1$ indicates that $v_{i}$ will go back to the isolated region after visiting the *j*th area of its sequence and $y_{i,j}\;=\;0$ otherwise. Consequently, a solution to the problem consists of both the set $X\;=\;\{x_{1},x_{2},\ldots ,x_{m}\}$ and set $Y\;=\;\{y_{1},y_{2},\ldots ,y_{m}\}$.

Nevertheless, the decision variables are not completely free. Based on experiences from the emergency management departments and related studies, we have a set of specific rules for cases where high-risk individuals in an area cannot be transferred by a vehicle in one round. Let $c_{\min}\;=\;\min_{1\leq i\leq m}c_{i}$, i.e., the minimum capacity among all vehicles. The first rule is as follows:

R1. If $b_{j}\;\leq \;c_{\min}$, then the area $a_{j}$ can only be assigned to one vehicle.

Note that the vehicle may still go to $a_{j}$ more than once, in the case when the vehicle goes to $a_{j}$ for the first time, it has already carried some individuals from other areas and its remaining capacity is less than $b_{j}$. In such a case, we have the second rule:

R2. If the area $a_{j}$ is only assigned to the vehicle $v_{i}$, then $v_{i}$ cannot go to the next area until all individuals in $a_{j}$ have been loaded.

In other words, if $v_{i}$ cannot transfer all individuals in $a_{j}$ in one round and there is no other vehicle assigned for $a_{j}$, then $v_{i}$ should go back and forth between $a_{j}$ and the isolated region until there is no individual left in $a_{j}$.

The third rule limits the number of vehicles that can be utilized to transfer high-risk individuals in an area:

R3. Let $k_{j}$ be a positive integer satisfying $\left(k_{j}\;-\;1\right)c_{\min}\;<\;b_{j}\;\leq \;k_{j}c_{\min}$, then the area $a_{j}$ can be assigned to at most $k_{j}$ vehicles.

When there are two or more vehicles scheduled to transfer high-risk individuals in an area, we have the fourth rule:

R4. If an area $a_{j}$ is assigned to two or more vehicles, then the vehicle first arrives at $a_{j}$ should load individuals in $a_{j}$ as much as possible in the first round.

That is if the available capacity of the vehicle first arrives at $a_{j}$ is not smaller than $b_{j}$, it should load all $b_{j}$ individuals; otherwise, should use up its capacity in the first round.

Finally, the fifth rule generalizes the application scope of the above four rules:

R5. Whenever a vehicle has been scheduled, the rules R1–R4 still apply to the remaining subproblem.

In general, we first determine the schedule of the vehicle with the minimum capacity without violating R1–R4, and then recalculate $c_{\min}$ of the remaining vehicles; this process recursively continues until all vehicles have been scheduled. For the convenience of applying these rules, without loss of generality, we assume that all vehicles in *V* have been sorted in nondecreasing order of $c_{i}$.

### 3.3. Solution Evaluation

As our objective is to reduce the transmission risk associated with the individuals during their exposure, we need to calculate the exposure duration of each high-risk individual, i.e., the time period before he/she is loaded into a quarantine vehicle. We first consider a simplified version where *each area is only assigned to one vehicle*. For each vehicle $v_{i}$, its arrival time at its first area $a_{x_{i,1}}$ is:

(1) $$\tau_{i,1}=t_{i,x_{i,1}}$$

The capacity of $v_{i}$ when it arrives at $a_{x_{i,1}}$ is:

(2) $$c_{i,1}=c_{i}$$

If $b_{x_{i,1}}\;\leq \;c_{i,1}$, then $v_{i}$ can load all $b_{x_{i,1}}$ individuals at a time and then leave the area; otherwise, according to the rule R2, $v_{i}$ should go back and forth between the area and isolated region for $k_{i,1}$ times, where

(3) $$k_{i,1}=\left\lfloor \frac{b_{x_{i,1}}}{c_{i}}\right\rfloor$$

Here, ⌊x⌋ takes the largest integer smaller than or equal to *x*, and ⌈x⌉ takes the largest integer larger than or equal to *x*.

Consequently, the time at which $v_{i}$ finally leaves $x_{i,1}$ is

(4) $$\tau_{i,1}^{\prime }\;=\;\left\{\begin{array}{cc} \tau_{i,1}\;+\;\left(b_{x_{i,1}}\;-\;1\right)\Delta t_{i,x_{i,1}}, & b_{x_{i,1}}\;\leq \;c_{i,1}\\ \tau_{i,1}\;+\;2k_{i,1}t_{i,x_{i,1}}^{\prime }\;+\;\left(b_{x_{i,1}}\;-\;1\right)\Delta t_{i,x_{i,1}}, & b_{x_{i,1}}\;>\;c_{i,1} \end{array}\right.$$

The arrival time and capacity at the next area can always be calculated based on the leaving time and capacity of the previous area:

(5) $$\tau_{i,j\;+\;1}\;=\;\left\{\begin{array}{cc} \tau_{i,j}^{\prime }+t_{i,x_{i,j},x_{i,j\;+\;1}}, & y_{i,j}\;=\;0\\ \tau_{i,j}^{\prime }+t_{i,x_{i,j}}^{\prime }+t_{i,x_{i,j\;+\;1}}^{\prime }, & y_{i,j}\;=\;1 \end{array}\right.$$

(6) $$c_{i,j\;+\;1}\;=\;\left\{\begin{array}{cc} c_{i,j}-\left(b_{x_{i,j}}\;\;\mathrm{mod}\;\;c_{i}\right), & y_{i,j}\;=\;0\\ c_{i}, & y_{i,j}\;=\;1 \end{array}\right.$$

The number of times that $v_{i}$ goes back and forth between the next area and isolated region depends on the intermediate decision and remaining capacity at the previous area:

(7) $$k_{i,j\;+\;1}\;=\;\left\{\begin{array}{cc} 0, & y_{i,j}\;=\;0\wedge b_{x_{i,j\;+\;1}}\;\leq \;c_{i,j}\\ 1+\lfloor \frac{b_{x_{i,j\;+\;1}}\;-\;c_{i,j}}{c_{i}}\rfloor , & y_{i,j}\;=\;0\wedge b_{x_{i,j\;+\;1}}\;>\;c_{i,j}\\ \lfloor \frac{b_{x_{i,j\;+\;1}}\;}{c_{i}}\rfloor , & y_{i,j}\;=\;1 \end{array}\right.$$

By analogy, the time at which $v_{i}$ finally leaves $x_{i,j}$ is

(8) $$\tau_{i,j}^{\prime }\;=\;\left\{\begin{array}{cc} \tau_{i,j}\;+\;\left(b_{x_{i,j}}\;-\;1\right)\Delta t_{i,x_{i,j}}, & b_{x_{i,j}}\;\leq \;c_{i,j}\\ \tau_{i,j}\;+\;2k_{i,j}t_{i,x_{i,j}}^{\prime }\;+\;\left(b_{x_{i,j}}\;-\;1\right)\Delta t_{i,x_{i,j}}, & b_{x_{i,j}}\;>\;c_{i,j} \end{array}\right.$$

The total transmission risk is evaluated as the sum of exposure durations of all high-risk individuals. For each area $a_{x_{i,j}}$, the exposure duration of the first individual is $\tau_{i,j}$, each next individual in the first round needs to wait another period of $\Delta t_{i,x_{i,j}}$, and the exposure duration of each individual in subsequent rounds should take the travel time of $v_{i}$ between the area and the isolated region into consideration. Consequently, the sum of exposure durations of individuals in the area can be calculated by Equation (9).

(9) $$T_{x_{i,j}}\;=\;\left\{\begin{array}{cc} b_{x_{i,j}}\left(\tau_{i,j}\;+\;\frac{b_{x_{i,j}}\;-\;1}{2}\Delta t_{i,x_{i,j}}\right), & b_{x_{i,j}}\;\leq \;c_{i,j}\\ b_{x_{i,j}}\left(\tau_{i,j}\;+\;\frac{b_{x_{i,j}}\;-\;1}{2}\Delta t_{i,x_{i,j}}\right)+k_{i,j}t_{i,x_{i,j}}^{\prime }\left(2b_{x_{i,j}}\;-\;\left(k_{i,j}\;+\;1\right)c_{i,j}\right), & b_{x_{i,j}}\;>\;c_{i,j} \end{array}\right.$$

The main difficulty lies in the case when *an area is assigned to two or more vehicles*. Let $A^{+}$ be the set of such areas and V(a) be the set of vehicles to which the area *a* is assigned. We use the following procedure to deal with this difficulty.

1. Initialize $T_{x_{i,j}}\;=\;0$ for all *i* and *j*, and initialize a set $A^{♭}\;=\;\left\{a_{x_{1,1}},a_{x_{2,1}},\ldots ,a_{x_{m,1}}\right\}$, i.e, $A^{♭}$ consists of the areas *to be first visited by the vehicles*.
2. Select from $A^{♭}$ the area $a^{*}$ with the earliest arrival time.
3. If $a^{*}\;\notin \;A^{+}$, i.e., all $b^{*}$ individuals can be loaded in one round, then use Equations (5)–(9) to calculate the sum of exposure durations of individuals in $a^{*}$ and the arrival time and capacity of the corresponding vehicle at the next area.
4. Otherwise, let $v_{i^{*}}$ be the first vehicle arriving at $a^{*}$, $b^{*}$ be the number of individuals in $a^{*}$, $j^{*}$ be the index of $a^{*}$ in the sequence of $v_{i^{*}}$, and $b^{\prime }\;=\;\min\left(b^{*},c_{i^{*}}\right)$, i.e., the number of individuals that can be loaded by $v_{i^{*}}$ in the current round. We first increase $T_{x_{i^{*},j^{*}}}$ by the exposure durations of individuals loaded by $v_{i^{*}}$ in this round:
  (10) $$T_{x_{i^{*},j^{*}}}\;=\;T_{x_{i^{*},j^{*}}}\;+\;b^{\prime }\left(\tau_{i^{*},j^{*}}\;+\;\frac{b^{\prime }\;-\;1}{2}\Delta t_{i^{*},x_{i^{*},j^{*}}}\right)$$
  and then update the number of remaining individuals in $a^{*}$ as
  (11) $$b^{*}\;=\;b^{*}-b^{\prime }$$
  (4.1) If $b^{*}\;>\;0$, temporally update the arrival time of $v_{i^{*}}$ after the current round of transfer:
    (12) $$\tau_{i^{*},1}\;=\;\tau_{i^{*},1}+\left(b^{\prime }\;-\;1\right)\Delta t_{i^{*},x_{i^{*},j^{*}}}+2t_{i,x_{i^{*},1}}^{\prime }$$
    and then go to step 2).
  (4.2) Otherwise $b^{*}\;=\;0$, use Equations (5) and (6) to calculate the arrival time and capacity of $v_{i^{*}}$ at the next area.
  (4.3) For each other $v_{i^{\prime }}\;\in \;V\left(a^{*}\right)$, restore its arrival time at $a^{*}$ to the value of the previous round (if exists, because after returning to the isolated region, $v_{i^{\prime }}$ can directly go to the next area instead of revisiting $a^{*}$), and use Equations (5) and (6) to calculate its arrival time and capacity at the next area.
5. Remove $a^{*}$ from both $A^{+}$ and $A^{♭}$; if $a^{*}$ is not the last area in the sequence, add the next area to $A^{♭}$.
6. Go to step 2) until $A^{♭}$ is empty.

**Figure 2:**
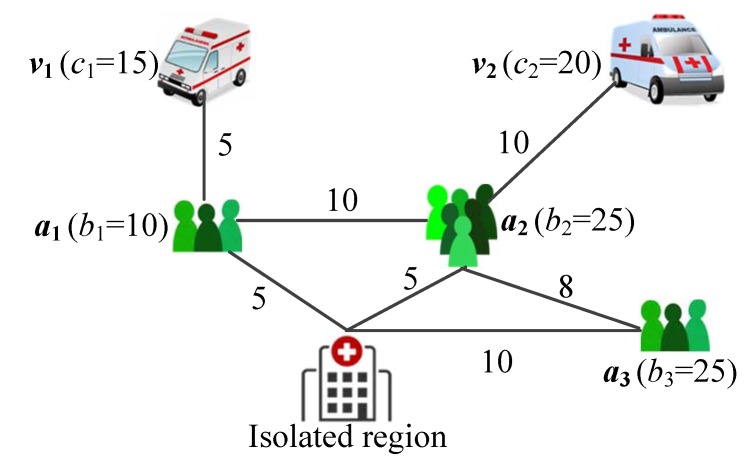
A simple problem instance with two vehicles and three areas. We assume that the vehicles have the same velocity, and the label on a line denotes the travel time between two areas; we also assume that the loading time interval $\Delta t_{j}$ is 0.5 for all three areas.

For illustration, consider a simple example shown in Figure 2. Suppose that $x_{1}=\left\{1,2,3\right\}$, $y_{1}=\left\{0,1\right\}$, $x_{2}=\left\{2,3\right\}$, $y_{2}=\left\{1\right\}$. According to the above procedure, $A^{♭}$ is initialized as $\left\{a_{1},a_{2}\right\}$, for which we have $\tau_{1,1}\;=\;5$ and $\tau_{2,1}\;=\;10$. In the first iteration, we select $a^{*}\;=\;a_{1}$ and $i^{*}\;=\;1$, and then calculate

$$\begin{array}{ccc} b^{\prime } & \;=\; & 10\\ T_{1} & \;=\; & 10(5\;\;+\;\;0.5(10\;-\;1)/2)\;=\;72.5\\ \tau_{1,1}^{\prime } & \;=\; & 5\;\;+\;\;0.5(10\;-\;1)=9.5\\ \tau_{1,2} & \;=\; & 9.5\;\;+\;\;10\;=\;19.5 \end{array}$$

In the second iteration, we have $A^{♭}\;=\;\left\{a_{2}\right\}$, $a^{*}\;=\;a_{2}$ and $i^{*}\;=\;2$, and then calculate

$$\begin{array}{ccc} b^{\prime }\; & = & \;20\\ T_{2}\; & = & \;15(10\;\;+\;\;0.5(20\;-\;1)/2)\;=\;221.25\\ \tau_{2,2}\; & = & \;10\;\;+\;\;0.5(20\;-\;1)+2\;\;\times \;\;5\;=\;29.5 \end{array}$$

In the third iteration, we select $a^{*}\;=\;a_{2}$ and $i^{*}\;=\;1$, and then calculate

$$\begin{array}{ccc} b^{\prime } & \;=\; & 5\\ T_{2} & \;=\; & 221.25\;\;+\;\;5(19.5\;\;+\;\;0.5(5\;-\;1)/2)\;=\;323.75\\ \tau_{1,2}^{\prime } & \;=\; & 19.5\;\;+\;\;0.5(5\;-\;1)\;=\;21.5\\ \tau_{1,3} & \;=\; & 21.5\;\;+\;\;5\;\;+\;\;10=\;36.5 \end{array}$$

As now $b^{*}=0$, we restore $\tau_{2,2}\;=\;10$ and recalculate $\tau_{2,2}^{\prime }\;=\;10\;\;+\;\;0.5\left(20\;-\;1\right)=\;19.5$ and $\tau_{2,3}^{\prime }\;=\;19.5\;\;+\;\;5\;\;+\;\;10=34.5$. Similarly, in the remaining iterations, individuals in $a_{3}$ are first transferred by $v_{2}$ followed by $v_{1}$.

The objective of the problem is to minimize the sum of exposure durations of all individuals, and hence the problem are formulated as

(13) $$\begin{array}{cc} \min & f\left(X,Y\right)=\sum_{j=1}^{n}T_{j}\\ s.t. & \mathrm{Equations}(1)–(12) \end{array}$$

(14) $$\begin{array}{c} \overset{m}{\underset{i=1}{⋃}}x_{i}=A \end{array}$$

(15) $$\begin{array}{c} 1\leq x_{i,j}\leq n,\;1\;\leq \;i\;\leq \;m;1\;\leq \;j\;\leq \;n_{i} \end{array}$$

(16) $$\begin{array}{c} y_{i,j}\in \left\{0,1\right\},\;1\;\leq \;i\;\leq \;m;1\;\leq \;j\;\leq \;n_{i}\;-\;1 \end{array}$$

Due to the inclusion of *Y* into the decision, the considered problem is significantly more complex than the basic VRP.

## 4. Method

In a large-scale epidemic, we often need to schedule multiple vehicles to transfer hundreds to thousands of high-risk individuals distributed in many areas. The solution space of such a problem instance is huge, for which exact algorithms such as branch-and-bound can be impractical, and most metaheuristics for VRPs also converge slowly and cannot produce a satisfactory solution within a short response time.

To efficiently solve the problem, we adopt the WWO metaheuristic [3], which uses a small-size population (typically of 5–10 solutions) and hence requires low computational resources. We adapt the operators of WWO to evolve solutions to the problem, and develop a gradual neighborhood search method to improve solution accuracy. Algorithm 1 presents the pseudocode of the algorithm framework and the following subsections describe its operators in detail.

**Algorithm 1:** The hybrid WWO and neighborhood search algorithm for the quarantine vehicle routing problem.

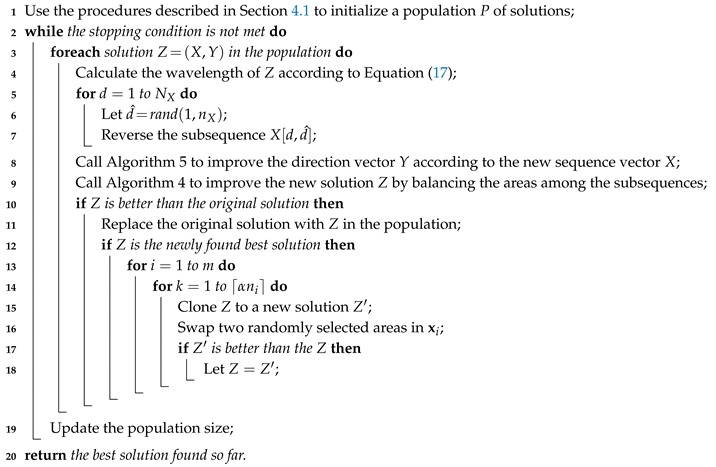



### 4.1. Solution Encoding, Initialization, and Improving

In the proposed hybrid algorithm, each solution is represented by two vectors, $X\;=\;\{x_{1},x_{2},\ldots ,x_{m}\}$ (which we call the sequence vector) and $Y\;=\;\{y_{1},y_{2},\ldots ,y_{m}\}$ (which we call the direction vector), each of which consists of *m* sub-vectors. To split two adjacent subsequences in *X*, we insert a symbol ‘0’ between them.

The algorithm starts by randomly initializing a population *P* of solutions. To create a random solution, we first generate *X* as a random permutation of *n* areas and then randomly select m−1 positions to insert separators ‘0’, and thus obtain *m* subsequence vectors for *m* vehicles. As an area may be assigned to multiple vehicles, we check *X* and select all areas satisfying $b_{x_{i,j}}\;<\;c_{i}$, the set of which is denoted by $A^{†}$. For each $a_{j}\;\in \;A^{†}$, let c(j) be the total capacity of vehicles to which $a_{j}$ has been assigned, we use $\left(b_{j}\;-\;c\left(j\right)\right)/b_{j}$ as the probability that $a_{j}$ will be assigned to more vehicles, and this probability-based assignment is recursively applied until there are no remaining individuals in $a_{j}$, as shown in Algorithm 2.

Next, we generate *Y* by using the procedure shown in Algorithm 3 to determine the direction $y_{i,j}$ on each area $x_{i,j}$ (1≤i≤m). That is, the probability of the vehicle $v_{i}$ goes from $a_{j}$ to the isolated region is proportional to its loading rate (a fully-loaded vehicle must go to the isolated region).

**Algorithm 2:** The procedure for assigning the individuals in an area to probably more than one vehicle.

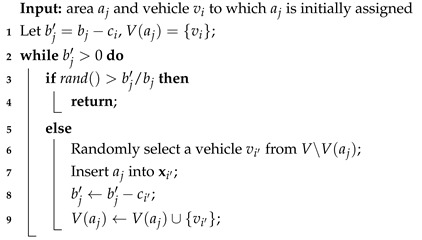



**Algorithm 3:** The procedure for generating the direction vector.

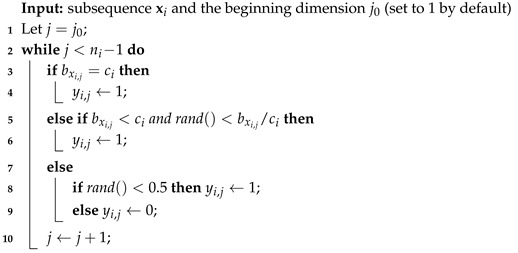



Usually, a randomly generated solution has much room for improvement. An obvious case is that when a vehicle has completed its job, another one still has many areas to visit. We use the greedy procedure shown in Algorithm 4 to tentatively improve a solution by continually moving an area from the sequence of the vehicle with the maximum completion time to that of the vehicle with the minimum completion time.

**Algorithm 4:** The procedure that improves a solution by balancing areas among vehicles.

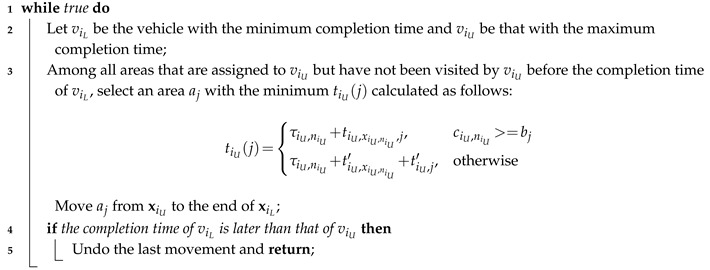



### 4.2. Water Wave Optimization for Evolving Solutions

WWO is a metaheuristic that takes inspiration from natural wave motions and has exhibited state-of-the-art performance in solving a variety of continuous, combinatorial, and constrained optimization problems [3,44,45]. In WWO, a solution is analogous to a wave, which has a wavelength inversely proportional to the solution fitness. The better (worse) the solution, the smaller (larger) the range in which the solution explores. As the problem is to minimize the objective function Equation (13), we employ the following equation to calculate the wavelength λ(Z) of each solution Z=(X,Y):

(17) $$\lambda \left(Z\right)=\frac{f(Z)}{\sum_{Z^{\prime }\in P}f\left(Z^{\prime }\right)}$$

The original WWO has three evolutionary operators: propagation, refraction, and breaking. Here, we adopt the strategy of improved WWO that discards refraction and reduces the population size with an increasing number of generations. We adapt the propagation operator to evolve a solution Z=(X,Y) to this problem as follows: let $n_{X}\;=\;\sum_{i=1}^{m}n_{i}$ be the total length of *X*, for each dimension $d\;<\;n_{X}$, with a probability of λ(Z), reverse a subsequence from dimension *d*, where the length of the subsequence is randomly set between $\left[1,n_{X}\;-\;d\right]$. Note that if the subsequence does not contain a separator, then the reversal only changes the route of one vehicle, but does not change the set of areas assigned to the vehicle; otherwise, it changes the routes of and reassigns the areas among two or more vehicles; in particular, after a reversal, it is probable that an area occurs in a subsequence more than once, and in such a case we remove the duplicates. The expected number of reversal on the solution is $\lambda \left(Z\right)n_{X}$, so a high (low) fitness solution will be changed to a short (large) extent. If the best resulting solution is better, it will replace the original one in the next generation. The pseudocode of the propagation operation is given in Lines 5–7 of Algorithm 1.

The breaking operator is conducted only on each newly found best solution $Z^{*}\;=\;\left(X^{*},Y^{*}\right)$. It tries to improve each subsequence $x_{i}^{*}$ by performing a local search for $\left\lceil \alpha n_{i}\right\rceil$ times, each swapping two randomly selected areas in $x_{i}^{*}$, where α is a control parameter less than 1. Among $\left\lceil \alpha n_{i}\right\rceil$ swaps, the best resulting one, if decreasing the total exposure time, is retained in $x_{i}^{*}$. The pseudocode of the breaking operation is given in Lines 14–19 of Algorithm 1.

### 4.3. Gradual Neighborhood Search for Direction Improving

The purpose of the gradual neighborhood search method is to fine-tune the direction vector *Y* of each solution Z=(X,Y). For each sub-vector $y_{i}$, the method gradually performs at most $\left(n_{i}\;-\;1\right)$ neighborhood search operations, each changing the decision $y_{i,j}$ (if the current capacity of $v_{i}$ allows) and then updating the subsequent dimensions from j+1 to $n_{i}\;-\;1$ according to the heuristic described in Algorithm 3. If the best neighboring sub-vector is better than the original sub-vector $y_{i}$, it will replace $y_{i}$ in the solution. Algorithm 5 presents the pseudocode of the gradual neighborhood search method.

**Algorithm 5:** The gradual neighborhood search method for improving the direction vector of a solution.

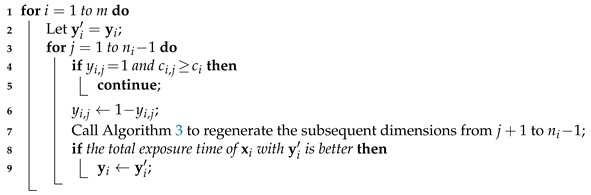



## 5. Results and Discussion

We test the proposed method on seven real-world instances of vehicle routing for transferring high-risk individuals in Hangzhou, China, from 28 January to 3 February 2020, the peak period of the COVID-19 epidemic in the city. Table 2 summarizes the basic characteristics of the seven instances.

To validate the performance of the hybrid WWO and neighbor search algorithm, we compare it with the following five methods:

- A greedy heuristic method, which always makes each vehicle move to the closest area with untransferred individuals. This method was used by most emergency management officers in the past.
- A standard integer programming algorithm implemented by CPLEX solver (version 12.6, with the automatic cut generator switched on).
- An ACO algorithm based on [19], which uses an artificial ant to construct and iteratively improve the tour of each vehicle.
- A memetic algorithm combing GA and local search for multi-trip VRP, in which a vehicle can go back to the depot and be re-loaded [46]. We adapt the algorithm for our problem by changing the objective function from the total travel time to the total exposure time.
- A tabu search algorithm for VRP with split deliveries and pickups [47].

The computational environment is a workstation with an i7-6500 2.5GH CPU, 8GB DDR4 RAM, and an NVIDIA Quadro M500M card. In each instance, as it is required to produce a solution within ten minutes, the maximum CPU time of each algorithm is set to 600 seconds. The last four algorithms are stochastic; in a real-world application, we typically run an algorithm once, or simultaneously run several instances of the algorithm with different random seeds and submit the best solution obtained to the decision-maker. However, to make the comparison more objective, we make up the number of runs of the ACO, memetic, tabu search, and WWO algorithms to 30 after the application.

**Figure 3:**
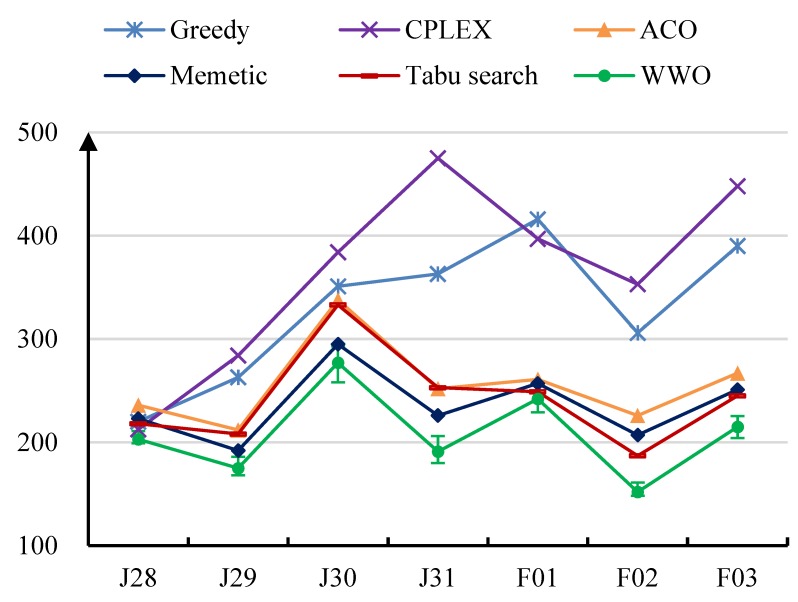
Comparative results of the algorithms on the test instances. The horizontal axis denotes the test instance, and the vertical axis denotes the average exposure time (in minutes).

Table 3 presents the results in terms of the average exposure time (in minutes) obtained by the algorithms on the test instances. For the last four algorithms, we present both mean value and standard deviation (in parenthesis). Figure 3 also illustrates the comparative results, where the maximum and minimum values obtained by WWO are given by error lines. As we can observe, the proposed algorithm achieves the shortest average exposure time (shown in bold in Table 3) on each instance. On instance J28 where the numbers of areas and high-risk individuals are relatively small, the differences among the algorithms are not significant, and the result of CPLEX is the second best. However, with an increasing number of areas/individuals, the performance of CPLEX becomes the worst among all algorithms on the six remaining instances. This is because, for large instances, the exact algorithm of CPLEX can only explore a very small fraction of the solution space within the limited running time. The ACO algorithm performs the worst on instance J28, but it outperforms Greedy and CPLEX on the remaining instances. The memetic algorithm performs the second-worst on J28, the second-best on J29, and the third-best (below tabu search and WWO) on the last four instances. This demonstrates that the population-based ACO and memetic metaheuristics can explore the large solution space in an efficient (if not the most efficient) way. The performance of the greedy heuristic is unstable, worse than that of CPLEX on J28 and F01 while better than that of CPLEX on the remaining instances. That is, on some specific instances, the greedy heuristic can sometimes reach a good solution, but its general performance is far from satisfactory. The tabu search algorithm starts from a greedy solution and then iteratively improves it. It performs relatively well on medium-size instances F01, F02, and F03; on small-size instances, its performance heavily relies on the unstable initial solution; on large instances, it cannot effectively explore the large solution spaces as the population-based metaheuristics. The average exposure time obtained by the greedy heuristic method used by the organization in the past is 337 minutes, while that of the proposed WWO-based algorithm is 206 minutes, which demonstrates that the proposed algorithm can significantly increase the efficiency of epidemic control.

**Figure 4:**
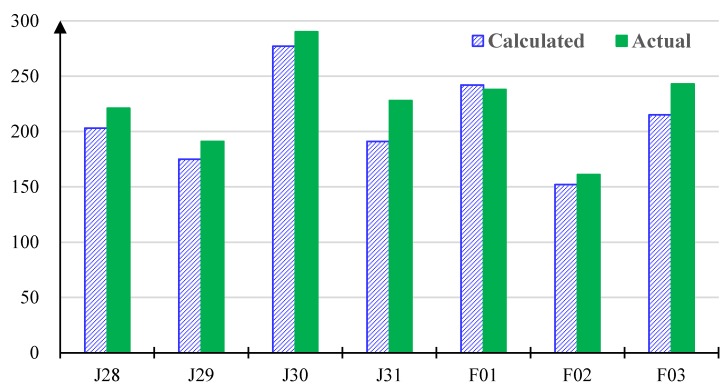
Comparison of the calculated and actual average exposure times on the test instances. The horizontal axis denotes the test instance, and the vertical axis denotes the average exposure time (in minutes).

Inevitably, the exposure time calculated based on the objective function of a solution is not equal to the actual exposure time obtained by the implementation of the solution. Thereby, we compare the calculated and actual exposure times on the test instances in Figure 4, from which we can observe that, except on F01 the calculated time is longer than the actual time, on each remaining instance the actual time is longer. This is because the solutions obtained by our algorithm have high qualities, i.e., they schedule the vehicles in an efficient and “compact” way; when a vehicle is delayed by an unexpected incident, many areas (especially those requiring multiple rounds of transfer) may be seriously affected, which can cause a significant increase of the total exposure time. Nevertheless, the calculated time does not deviate much from the actual time, which demonstrates the feasibility and accuracy of the solution produced by our approach.

In summary, the application of our method can be divided into the following steps.

(1) In preparation for an epidemic, establish a database to manage the information of quarantine vehicles, services for medical isolation, and routes from most densely populated areas to the isolated regions;
(2) Whenever there are requirements for high-risk individual transfer, input the number and distribution of individuals, and run the algorithm;
(3) Arrange staffs to prepare the vehicles (typically, during the execution of the algorithm);
(4) Within the response time, take the best-known solution found by the algorithm, and send it to the staffs for implementation;
(5) Monitor the operations; if the implementation deviates from the plan, or new requirement comes, adjust the current solution or run the algorithm to solve the new instance.

In practice, we must pay attention to the changes in information, including traffic conditions, vehicle conditions, and the number of high-risk individuals, and appropriately respond to the changes. For example, if the number of individuals in an area increases a small amount during the implementation of an existing solution, we typically make the vehicle for this area to transfer the new individuals and, if the vehicle is delayed too much, the last area(s) of the vehicle can be reassigned to other vehicle(s) that have completed their jobs. However, if the number of individuals increases a large amount, we often need to construct and solve a new instance for the remaining tasks.

## 6. Conclusions

Transferring high-risk individuals to an isolated region in a timely manner is critical for epidemic control. In this paper, we present a problem of scheduling quarantine vehicles to transfer high-risk individuals from a set of dispersed areas to an isolated region, which is more difficult than most existing VRPs. To solve this problem, we propose a hybrid WWO and neighborhood search algorithm, which adapts the WWO metaheuristic to efficiently explore the solution space and utilizes neighborhood search to improve the solution accuracy. Computational results demonstrate that the proposed algorithm significantly outperforms several existing algorithms and obtains high-quality solutions on the test instances.

Currently, our problem assumes that available drivers for quarantine vehicles are sufficient. During the peak period of COVID-19, we found that this assumption holds for the instances in Hangzhou and many other cities in China, but does not hold for that in Wuhan, the epidemic center where the number of high-risk individuals is huge and hence a vehicle should have several drivers in rotation. Therefore, an ongoing study is to include the scheduling of drivers in our problem. Our future work will integrate more other components, such as combining the use of electric and fuel vehicles [48], early primary screening and automatic grading of individuals [49], to enhance the effectiveness of epidemic control.

## Figures and Tables

**Table 1:** Mathematical variables used in the problem formulation.

Symbol	Description
*A*	Set of areas with high-risk individuals
*n*	Size of *A*
$a_{j}$	*j*th area in *A* (1≤j≤n)
$b_{j}$	Number of individuals in $a_{j}$
*V*	Set of vehicles
*m*	Size of *V*
$v_{i}$	*i*th vehicle in *V* (1≤i≤m)
$c_{i}$	Capacity of $v_{i}$
$c_{\min}$	Minimum capacity among all vehicles
$t_{i,j}$	Travel time of $v_{i}$ from its original location to $a_{j}$
$t_{i,j,j^{\prime }}$	Travel time of $v_{i}$ from $a_{j}$ to $a_{j^{\prime }}$
$t_{i,j}^{\prime }$	Travel time of $v_{i}$ from $a_{j}$ to the isolated region
$\Delta t_{i,j}$	Average time interval between loading two individuals in $a_{j}$ by $v_{i}$
$x_{i}$	Sequence of areas assigned to $v_{i}$
$x_{i,j}$	*j*th area in $x_{i}$
$y_{i}$	Sequence of intermediate decisions of $v_{i}$
$y_{i,j}$	Boolean variable denoting whether $v_{i}$ goes back to the isolated region
	after loading individuals in $x_{i,j}$
*X*	Vector of sequences $\left\{x_{1},\ldots ,x_{i},\ldots ,x_{m}\right\}$
*Y*	Vector of sequences $\left\{y_{1},\ldots ,y_{i},\ldots ,y_{m}\right\}$
$\tau_{i,j}$	First arrival time of $v_{i}$ at $x_{ij}$
$\tau_{i,j}^{\prime }$	Final leave time of $v_{i}$ at $x_{ij}$
$c_{i,j}$	Remaining capacity of $v_{i}$ when it arrives at $x_{ij}$
$k_{i,j}$	Number of times that $v_{i}$ goes back and forth between $a_{j}$ and isolated region
$A^{+}$	Set of areas, each of which has been assigned to two or more vehicles
V(a)	Set of vehicles to which the area *a* is assigned
$T_{j}$	Total exposure time of individuals in $a_{j}$

**Table 2:** Summary of the basic characteristics of the seven real-world instances. $\bar{b}$: average number of individuals per area; $K_{V}$: number of types of vehicles; $\bar{c}$: the average capacity of vehicles; $\bar{t}$: average travel time between two areas (in minutes).

ID	*n*	*m*	$\bar{b}$	$K_{V}$	$\bar{c}$	$\bar{t}$
J28	27	6	13.9	2	11.0	44.5
J29	60	9	10.8	2	11.0	71.3
J30	127	16	10.4	4	10.5	60.7
J31	191	16	8.4	4	10.5	35.7
F01	93	15	10.6	4	10.8	54.2
F02	102	15	10.3	4	10.8	39.3
F03	98	12	8.2	4	9.3	48.2

**Table 3:** Results of the comparative algorithms on the test instances.

ID	Greedy	CPLEX	ACO	Memetic	Tabu Search	WWO
J28	220	213	236 (14.19)	223 (5.11)	218 ( 8.16)	**203** (5.87)
J29	263	284	212 (20.75)	192 (3.88)	208 ( 9.15)	**175** (7.20)
J30	351	384	338 (24.52)	295 (10.97)	333 (16.25)	**277** (13.49)
J31	363	475	252 (18.39)	226 (12.73)	253 (18.20)	**191** (9.28)
F01	416	397	261 (23.30)	257 (15.45)	249 (17.03)	**242** (10.07)
F02	306	353	226 (16.06)	207 (14.66)	187 (15.05)	**152** (5.25)
F03	390	448	267 (20.40)	251 (17.80)	245 (28.46)	**215** (6.70)

## Abbreviations

The following abbreviations are used in this manuscript:

VRP	vehicle routing problem
COVID-19	novel coronavirus pneumonia
GA	genetic algorithm
ACO	ant colony optimization
WWO	water wave optimization
